# Genetic diversity and natural selection in the rhoptry-associated protein 1 (RAP-1) of recent *Plasmodium knowlesi* clinical isolates from Malaysia

**DOI:** 10.1186/s12936-016-1127-7

**Published:** 2016-02-05

**Authors:** Mira Syahfriena Amir Rawa, Mun-Yik Fong, Yee-Ling Lau

**Affiliations:** Faculty of Medicine, Department of Parasitology, University of Malaya, 50603 Kuala Lumpur, Malaysia; Tropical Infectious Diseases Research and Education Centre (TIDREC), University of Malaya, 50603 Kuala Lumpur, Malaysia

**Keywords:** *Plasmodium knowlesi*, Rhoptry-associated protein 1, Genetic diversity, Selection, Haplotypes

## Abstract

**Background:**

The *Plasmodium* rhoptry-associated protein 1 (RAP-1) plays a role in the formation of the parasitophorous vacuole following the parasite’s invasion of red blood cells. Although there is some evidence that the protein is recognized by the host’s immune system, study of *Plasmodium falciparum* RAP-1 (PfRAP-1) suggests that it is not under immune pressure. A previous study on five old (1953–1962) *P. knowlesi* strains suggested that RAP-1 has limited genetic polymorphism and might be under negative selection. In the present study, 30 recent *P. knowlesi* isolates were studied to obtain a better insight into the polymorphism and natural selection of *Pk*RAP-1.

**Methods:**

Blood samples from 30 *knowlesi* malaria patients were used. These samples were collected between 2010 and 2014. The *PkRAP-1* gene, which contains two exons, was amplified by PCR, cloned into *Escherichia coli* and sequenced. Genetic diversity and phylogenetic analyses were performed using MEGA6 and DnaSP ver. 5.10.00 programs.

**Results:**

Thirty *Pk*RAP-1 sequences were obtained. The nucleotide diversity (π) of exons 1, 2 and the total coding region (0.00915, 0.01353 and 0.01298, respectively) were higher than those of the old strains. Further analysis revealed a lower rate of non-synonymous (d_N_) than synonymous (d_S_) mutations, suggesting negative (purifying) selection of *Pk*RAP-1. Tajima’s D test and Fu and Li’s D test values were not significant. At the amino acid level, 22 haplotypes were established with haplotype H7 having the highest frequency (7/34, 20.5 %). In the phylogenetic analysis, two distinct haplotype groups were observed. The first group contained the majority of the haplotypes, whereas the second had fewer haplotypes.

**Conclusions:**

The present study found higher genetic polymorphism in the *PkRAP-1* gene than the polymorphism level reported in a previous study. This observation may stem from the difference in sample size between the present (n = 30) and the previous (n = 5) study. Synonymous and non-synonymous mutation analysis indicated purifying (negative) selection of the gene. The separation of *Pk*RAP-1haplotypes into two groups provides further evidence to the postulation of two distinct *P. knowlesi* types or lineages.

**Electronic supplementary material:**

The online version of this article (doi:10.1186/s12936-016-1127-7) contains supplementary material, which is available to authorized users.

## Background

The pathogenesis of malaria parasites incorporates the orchestrated action of various proteins, a few of which are primary targets for anti-malarial vaccines. These proteins frequently exhibit high levels of heterozygosity, and their rapid rates of evolution may be essential for the parasite to escape the host’s immune defence [1]. Highly polymorphic proteins are often favoured by positive selection, in which selective forces, such as immune responses and drugs, drive the genes expressing these antigenic proteins to accumulate mutations and maintain them in the population [2]. This strategy enables the parasite to manifest antigenically different alleles to thwart the host’s immune response. Alternatively, these alleles may be eliminated or negatively selected in the case of less fit genetic variants.

The *Plasmodium* merozoite invasion of red blood cells involves binding, apical orientation and secretion of apical organelle contents known as rhoptries, micronemes and dense granules [3–5]. Proteins in these organelles have been implicated in key aspects of invasion. These include the formation of moving junctions between the merozoite and erythrocyte surfaces, which subsequently leads to the formation of the parasitophorous vacuole in which the parasite resides. Rhoptry-associated protein 1 (RAP-1) plays a role in the latter process [3], although its precise function is unknown. RAP-1 forms a complex with smaller proteins, RAP-2 or RAP-3, and deletion of the *RAP-1* gene results in mistargeting of RAP-2 to the rhoptries [6].

Limited polymorphism in the *Plasmodium falciparum* RAP-1 (*Pf*RAP-1) suggests that it is not under an immune pressure [7]. However, there is some evidence that RAP-1 is recognized by the host’s immune system and that antibodies to this protein inhibit merozoite invasion [7]. For example, monoclonal antibodies against *Pf*RAP-1 hindered erythrocyte invasion in vitro [8, 9] and partial protection against *P. falciparum* challenge infection was observed in *Saimiri sciureus* and *S. boliviensis* monkeys immunized with *Pf*RAP-1 and *Pf*RAP-2 [10, 11]. Although there have been extensive studies of *Pf*RAP-1, studies on the *P. knowlesi* orthologue are limited.

In a recent investigation it was demonstrated that negative selection might be acting on the RAP-1 of non-human primate parasites, including *P. knowles*i [7]. However, the study used only five old (isolated in 1955–1965) *P. knowles*i strains, which may not reflect the true picture of polymorphism in *P. knowles*i RAP-1 (*Pk*RAP-1). In the present study, the RAP-1 of 30 recently isolated *P. knowlesi* was investigated to obtain a better picture of the parasite’s diversity.

## Methods

### Blood sample collection and ethics approval

Between 2010 and 2014, 30 blood samples of patients with *P. knowlesi* infection were collected from the University of Malaya Medical Centre and several private clinics in Peninsular Malaysia. Ethics approval for the use of the blood samples was granted by the University of Malaya Medical Centre Ethic Committee (MEC No. 817.18). *P. knowlesi* infection in each patient was confirmed by microscopic examination of Giemsa-stained thin and thick blood smears and polymerase chain reaction (PCR) amplification using diagnostic primers [12].

### Extraction of DNA

*Plasmodium knowlesi* genomic DNA was extracted from 100 μl of each blood sample using the QIAGEN Blood DNA Extraction Kit (QIAGEN, Hilden, Germany) following the manufacturer’s protocol. Extracted DNA was eluted in 100 μl of elution buffer.

### Amplification by PCR of *Pk*RAP-1

Amplification of the *PkRAP-1* gene was conducted by PCR using specific oligonucleotide primers *Pk*RAP-1F: 5′-CGT TGA GCA GGA AAT GCC TAC TCC AAT C-3′ and *Pk*RAP-1R: 5′-ATG ATA ACG TAC GCA AGT TCT CTG CTG G-3′. These primers (nucleotide positions 1782248–1782275 and 1784654–1784681) were based on the *RAP-1* gene sequence of *P. knowlesi* strain H (GenBank Accession No. AM910995). The high fidelity DNA polymerase GoTaq® Long PCR Mastermix (Promega, Madison, WI, USA) was used to provide proofreading activity and efficient long DNA amplification. PCR was conducted in a total volume of 25 ml that included a final concentration of 1 × PCR mastermix, 0.4 mM of each primer and 100–500 ng of total genomic DNA. Thermal cycling profile began with an initial denaturation step at 95 °C for 2 min, followed by 35 cycles at 94 °C for 30 s and 63 °C for 2 min and 30 s, with a final extension at 72 °C for 10 min. A PCR product with an expected size of 2433 or 2434 bp was detected following electrophoresis on 1 % agarose gels.

### Purification of PCR product and DNA cloning

Purification of PCR products was performed using the QIAquick PCR Purification Kit (QIAGEN) according to the manufacturer’s instructions. The concentration and purity of each product were determined using the NanoDrop 2000 (Thermo Fisher Scientific, Waltham, MA, USA). The purified PCR products were then ligated into the pGEM-T vector (Promega) and transformed into *Escherichia coli* TOP10F’ competent cells. Recombinant plasmids from the transformants were selected and sent to a commercial laboratory for DNA sequencing. To verify the sequences, the recombinant plasmids of three clones from each isolate were sequenced. In addition, the sequencing was performed in both directions of the inserts in the plasmids.

### Sequence and phylogenetic analyses

**Fig. 1:**
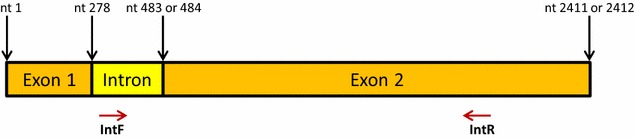
Schematic diagram of the *PkRAP-1* gene. Locations of exon 1, intron and exon 2 are shown. Locations of internal sequencing primers are also shown. IntF sequencing primer annealed at nucleotide positions 286–305, while IntR annealed at positions 2002–2021

In addition to the universal M13 sequencing primers, two internal primers, *Pk*RAP-1 IntF: 5′-ATG AGC AAA CCG TTC GTG TG-3′ and *Pk*RAP-1 IntR: 5′-GTG CAT ACT GGA AAG CAT GG-3′ were used for DNA sequencing to obtain the full-length *P. knowlesi RAP-1* gene sequence (Fig. 1). Each sequence was trimmed, joined and aligned using the AliView program. Thirty *Pk*RAP-1 sequences were obtained and aligned together with sequences of the Nuri strain (GenBank Accession No. GQ2816500, as the reference sequence), Hackeri strain (GenBank Accession No. GQ281651), Malayan strain (GenBank Accession No. GQ281648) and Philippines strain (GenBank Accession No.GQ281652). Both the nucleotide and deduced amino acid sequences were analysed using the CLUSTAL-Omega program [13]. The phylogenetic tree was constructed using the neighbour-joining method in MEGA6 [14]. When constructing the tree, bootstrap proportions of 1000 replicates were utilized to verify the robustness of the tree. *P. coatneyi* RAP-1 isolate (GenBank Accession No. GQ281653) was used as outgroup.

### RAP-1 sequence polymorphism analysis

The number of segregating sites (S), the number of haplotypes (H), haplotype diversity (Hd) and nucleotide diversity (π) were calculated using DnaSP version 5.10.00 [15]. To estimate the step-wise diversity across the *Pk*RAP-1, π was established on a sliding window of 100 bases, with a step size of 25 bp. The Z test (P < 0.05) in MEGA6, employing the Nei and Gojobori method and the Jukes and Cantor correction, was used to estimate and compare the rates of synonymous (d_S_) and non-synonymous (d_N_) substitutions. d_N_ will be lower than d_S_ (d_N_/d_S_ < 1) when a gene is under negative (purifying) selection, while d_N_ will be greater than d_S_ (d_N_/d_S_ > 1) when the positive selection is more advantageous. Tajima’s D [16] and Fu and Li’s D [17] test statistics in the DnaSP version 5.10.00 were used to detect departure from the neutral theory of evolution.

## Results

### Nucleotide diversity and genetic differentiation

The in *Pk*RAP-1 contains two exons and one intron (Fig. 1). PCR amplification using the above primers produced a fragment of either 2433 or 2434 bp fragment. The difference in the fragment size was due to the presence of an additional nucleotide in the intron of some of the isolates. After sequencing, the sequences trimmed to obtain the full length *Pk*RAP-1 (2411 or 2412 bp). Thirty sequences of *Pk* RAP-1 were obtained (GenBank Accession Numbers listed in Additional file 1). These sequences were aligned and analysed for the diversity and natural selection. A comparison was also made between these sequences and the *Pk* RAP-1 of old strains including Nuri, Hackeri, Malayan and Philippines (isolated in 1953, 1960, 1962, and 1961, respectively).

The results of the genetic diversity and neutrality tests of the *Pk*RAP-1 are presented in Table 1. The Hd for exon 1, exon 2 and the total coding region was 0.818, 0.993 and 0.995, respectively. Additionally, the nucleotide diversity (π) of exon 1, 2 and the total coding region was 0.00915, 0.01353 and 0.01298, respectively. Higher π values were observed in exon 2 and total coding region of the recent isolates compared to the corresponding π values of the old strains (exon 2: 0.0076; total coding region: 0.0082) [7]. However, there was not much difference between the π values of exon 1 of the old strains (0.0123) and recent isolates (0.00915). Interspecies comparison (Table 2) showed that the nucleotide diversity of *Pk*RAP-1was 3-fold higher than of *Pf*RAP-1 [7] and 14-fold higher than of *Pv*RAP-1 [18].

**Table 1:** Estimates of DNA diversity, selection, and neutrality tests of *Pk*RAP-1 in Malaysia

*Pk*RAP-1	n	S	Hd ± SD	π ± SD	d_N_ ± SE	d_S_ ± SE	d_N_/d_S_	Z test	Tajima’s D	Fu and Li’s D
Exon 1	34	276	0.818 ± 0.054	0.00915 ± 0.00089	0.00574 ± 0.00352	0.02253 ± 0.01104	0.25477	d_N_ = d_S_	−0.44307 (P > 0.10)	−0.47531 (P > 0.10)
Exon 2	34	1929	0.993 ± 0.009	0.01353 ± 0.00102	0.00894 ± 0.00145	0.03274 ± 0.00591	0.27306	d_N_ < d_S_ (P < 0.05)	−0.20877 (P > 0.10)	−0.22130 (P > 0.10)
Total CDS	34	2205	0.995 ± 0.009	0.01298 ± 0.00091	0.00854 ± 0.00126	0.03137 ± 0.00483	0.27223	d_N_ < d_S_ (P < 0.05)	−0.23957 (P > 0.10)	−0.26919 (P > 0.10)

*n* number of sequences, *S* number of sites, *Hd* haplotype diversity, π observed average pairwise nucleotide diversity, *d*_*N*_ rate of non-synonymous substitutions per non-synonymous site, *d*_*S*_ rate of synonymous substitutions per synonymous site

**Table 2:** Nucleotide diversity among the RAP-1 of *Plasmodium* species

Species	N	S	π	Reference
*P. falciparum*	32	2346–2349	0.0041	[7]
*P. vivax*	29	2413	0.00088	[19]
*P. knowlesi*	34	2411–2412	0.01298	Present study

*n* number of isolates, *S* number of sites, π nucleotide diversity

**Fig. 2:**
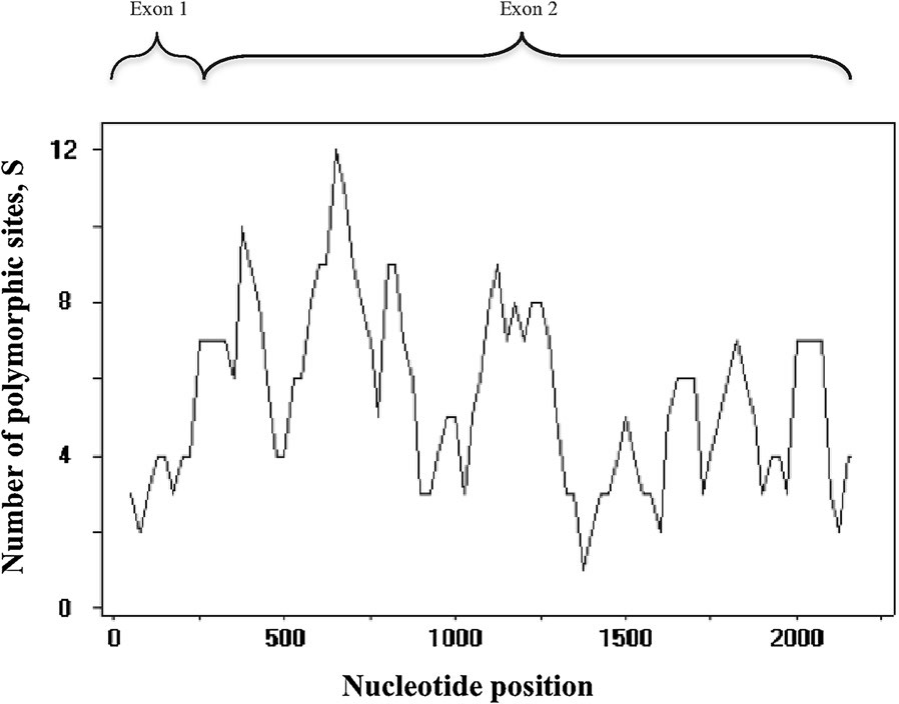
Nucleotide polymorphism of *Pk*RAP-1 Sliding window plot of number of polymorphic sites (S) in the *Pk*RAP-1 coding regions. The S values were calculated using DnaSP ver. 5.10.00 with a window length of 100 bp and a step size of 25 bp

The sliding window plot (window length 100 bp, step size 25 bp) revealed that exon 2 contained both the highest and lowest polymorphic regions (Fig. 2). The greatest diversity was observed within nucleotide positions 250–500 of the coding region, while the most conserved region was seen at nucleotide positions 1800–1950. The overall nucleotide diversity ranged from 0.003 to 0.033.

### Amino acid changes and phylogenetic analysis

**Fig. 3:**
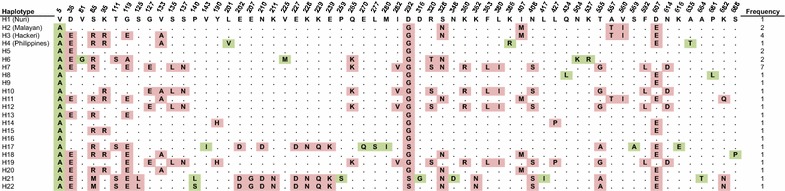
Amino acid sequence polymorphism in *Pk*RAP-1. Alignment of polymorphic amino acid residues showing 22 *Pk*RAP-1 haplotypes of the recent Malaysian *P. knowlesi* isolates and old strains. Haplotypes H1, H2, H3 and H4 are of the Nuri, Malayan, Hackeri and Philippines strains, respectively. The singleton sites are marked in *green* and the parsimony-informative sites are marked in *red*. Amino acid residues identical to those of Nuri strain are marked by *dots*. Frequency of each haplotype is listed in the *right panel*

A total of 735 amino acid residues were deduced from the *Pk*RAP-1 total coding region. Using the Nuri strain sequence as reference, 61 segregating sites were identified. Singleton sites were found to be lower in frequency (23/61) than the parsimony-informative sites (38/61). From these variable sites, 54 of them were dimorphic and seven were trimorphic changes (85 = R, M; 119 = E, A; 140 = L, S; 292 = G, S; 320 = S, T; 555 = G, A; 682 = N, Q) (Fig. 3).

**Fig. 4:**
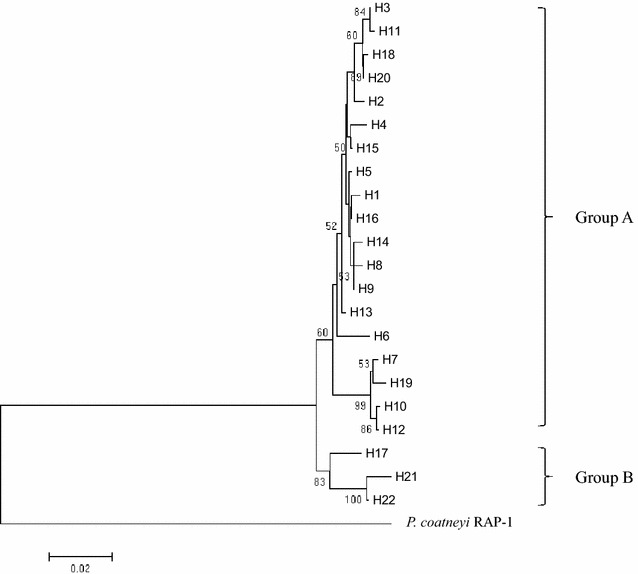
Phylogenetic tree of *Pk*RAP-1 haplotypes in Malaysia. Neighbour-joining phylogenetic tree of 22 haplotypes of *P. knowlesi* RAP-1, showing with two distinct groups, A and B. Numbers at nodes indicate percentage support of 1000 bootstrap replicates. *P. coatneyi* RAP-1 is used as outgroup

Twenty-two haplotypes were deduced from the amino acid sequences (Fig. 3). Haplotype H7 had the highest frequency (7/34, 20.5 %), followed by haplotype H3 (4/34, 11.76 %), and haplotypes H2, H5 and H6 (each 2/24, 5.88 %). It is interesting to note that some haplotypes consisted of old and recent isolates (Table 3). For instance, haplotype H2 contained the Malayan strain (1962) and isolate NG (2011). The Hackeri strain (1960) and three recent isolates (UM 0004, UM 0016 and UM 0092; isolated 2012–2013) were of haplotype H3. Phylogenetic tree analysis revealed that the haplotypes could be clustered into two main groups: A and B (Fig. 4). Group A consisted of 19 haplotypes, whereas Group B had three haplotypes. The haplotypes (H1–H4) of the four old strains were grouped together with those of the recent isolates in Group A.

**Table 3:** RAP-1 haplotypes of *Plasmodium knowlesi* strains and isolates

Haplotypes	Strain/isolate (year isolated)
H1	Nuri (1953)
H2	Malayan (1962), NG (2011)
H3	Hackeri (1960), UM 0004 (2012), UM 0016 (2012), UM 0092 (2013)
H4	Philippines (1961)
H5	UM 0002 (2012), UM 0115 (2014)
H6	MAI (2010), UM 0088 (2013)
H7	AZL (2011), UM 0006 (2012), UM 0018 (2012), UM 0047 (2013), UM 0050 (2013), UM 0058 (2013), UM 0060 (2013)
H8	ISM (2011)
H9	UM 0001 (2012)
H10	UM 0009 (2012)
H11	UM 0014 (2012)
H12	UM 0015 (2012)
H13	UM 0020 (2012)
H14	UM 0021 (2012)
H15	UM 0029 (2012)
H16	UM 0032 (2012)
H17	UM 0034 (2012)
H18	UM 0063 (2013)
H19	UM 0070 (2013)
H20	UM 0090 (2013)
H21	UM 0105 (2014)
H22	UM 0118 (2014)

### Natural selection in the *PkRAP-1* gene

A significant excess of synonymous substitutions was seen in the *Pk*RAP-1. The calculated ratios d_N_/d_S_ for exon 1, exon 2 and total coding region less than 1 (Table 1). This was indicative of negative selection of *Pk*RAP-1. Detailed analysis using the Z test revealed negative selection in exon 2, but neutral selection in exon 1. In the Tajima’s D and Fu and Li’s D tests, all values obtained for *Pk*RAP-1 were negative, but did not differ statistically (P > 0.10) significantly from zero. Therefore, Tajima’s D and related statistics did not detect departure from neutrality.

## Discussion

A study has been carried out previously on the diversity and natural selection of *Pk*RAP-1, albeit using a small sample size (n = 5) of old *P. knowlesi* strains [7]. The present study was carried out using the same approach, but using a larger sample size (n = 30) consisting of recent isolates. Unlike the findings on the old strains [π: 0.0082 (total coding region), 0.0123 (exon 1), 0.0076 (exon 2)], the present study found relatively higher diversity among the *Pk*RAP-1 of the recent isolates [π: 0.01298 (total coding region)], and diversity was much higher in exon 2 (π: 0.01353) than in exon 1(π: 0.00915). However, both the old strains and recent isolates showed negative selection in exon 2 and neutral selection in exon 1. The *Pk*RAP-1 (π: 0.01298) was observed to be relatively more diverse than *Pf*RAP-1 (π: 0.0041) [7] and *Pv*RAP-1 (π: 0.00088) [18]. A similar finding was reported for rhoptry bulb proteins [5]. It has been suggested that such contrasting level of polymorphism in rhoptry-related proteins is expected because these proteins are distinct across the *Plasmodium* species, presumably for adaptation in their respective target host cells [5].

Merozoite surface protein-8 (MSP-8), MSP-9, apical membrane antigen-1 (AMA-1) and Duffy binding protein (DBPαII) are among the widely studied proteins known to be potential vaccine candidates. For *P. knowles*i, the MSP-8 [19], MSP-9 [20] and AMA-1 [21] expressed lower genetic diversity (π: 0.0008 and 0.00501, respectively) than *Pk*RAP-1. *Pk* DBPαII (π: 0.013) [22], however, has almost similar diversity level with *Pk*RAP-1. Similar to *Pk*RAP-1, these proteins also appear to be under negative selection.

The sliding window plot analysis showed that *Pk* RAP-1 was more conserved at the C-terminal region. This is most likely due to the role of this region in a key binding activity. The RAP-1 is known to bind to RAP-2 or RAP-3 via its C-terminal region [6]. Furthermore, deletion of the RAP-1 C-terminus leads to RAP-1 mislocalization to the rhoptry neck instead of the bulb [3], suggesting the importance of this region in protein targeting. In contrast, the N-terminal of *Pk*RAP-1 exhibited genetic diversity and this may be due to the presence of T cell epitopes. It has been observed that lymphocytes gave response to the N-terminus of *Pf*RAP-1 [23, 24].

Many of the malaria parasite blood stage antigens, such as the merozoite surface proteins, display polymorhism as a result of positive selection [25]. This is said to be an escape mechanism for the parasite to evade the immune responses of the host. Antigenic polymorphism involving the expression of different alleles of the gene would hamper the host’s immune system to recognize the protein [2]. Immune defences, such as antibodies and T cells, will not be able to identify antigenically different epitopes, and these mutated alleles will then be selectively expanded. Negative selection usually minimizes genetic variants, therefore leading to low frequency rare alleles in the population. Low frequency rare haplotypes were evident among the *Pk*RAP-1 in the present study (Fig. 3).

Interestingly, negative selection is also seen in the RAP-1 gene of several non-human primate malarial parasites such as *P. cynomolgi, P. inui* and *P. fieldi* but not in human malaria parasites, such as *P. falciparum* and *P. vivax* [7]. For *P. knowles*i, this negative selection may be due to a bottleneck event that drives population expansion or growth. Mitochodrial DNA analysis have shown that *P. knowlesi* in Southeast Asia underwent significant population expansion approximately 30,000–40,000 years ago [26]. An alternative explanation for the negative selection is that *Pk*RAP-1, being an important protein in erythrocyte invasion, has functional constraints that limit polymorphism, and any variant form of *Pk*RAP-1 will be disadvantageous to the parasite.

The phylogenetic tree in this present study also showed separation of the *Pk*RAP-1 haplotypes into two groups (Fig. 4). This separation of *Pk*RAP-1 haplotypes groups may indicate dimorphism of the gene. Similar observations have been reported in *P. knowlesi* genes such as *Pk*DBPαII [22], *Pk*nbpxa [27], *Pk*AMA-1 domain I [28] and *Pk*MSP-1 [29]. These findings provide support to the postulation of the existence of two distinct *P. knowlesi* types or lineages in Southeast Asia [30]. Microsatellite genotyping data revealed admixture of two highly divergent *P. knowlesi* populations, and each population is associated with different forest-dwelling macaque reservoir host species [31]. Recently, a whole-genome population study showed two major sub-groups of *P. knowlesi* clinical isolates [32].

## Conclusions

The present study found higher genetic polymorphism in the *PkRAP-1* gene than the polymorphism level reported in a previous study. This observation may stem from the difference in sample size between the present (n = 30) and the previous (n = 5) study. Synonymous and nonsynonymous mutation analysis indicated purifying (negative) selection of the gene. The separation of *Pk*RAP-1 haplotypes into two groups is further evidence to the existence of two distinct *P. knowlesi* types or lineages.

## Additional file

10.1186/s12936-016-1127-7GenBank Accession Number of *Pk*RAP-1 sequences.
