# Supplementary material for: Genetic diversity and natural selection in the rhoptry-associated protein 1 (RAP-1) of recent Plasmodium knowlesi clinical isolates from Malaysia
Source: Malar J. 2016 Feb 5;15:62. doi: 10.1186/s12936-016-1127-7 (PMC4743133; doi:10.1186/s12936-016-1127-7)
Supplement: Supplementary file 1 — 10.1186/s12936-016-1127-7GenBank Accession Number of PkRAP-1 sequences. [file 12936_2016_1127_MOESM1_ESM.docx]

**GenBank Accession Number of *Pk*RAP-1 sequences**

| Isolate | Gene Accession No. |
| --- | --- |
| AZL | KR259544 |
| ISM | KR259548 |
| MAI | KR259552 |
| NG | KR259557 |
| UM 0001 | KR259562 |
| UM 0002 | KR259565 |
| UM 0004 | KR259567 |
| UM 0006 | KR259570 |
| UM 0009 | KR259572 |
| UM 0014 | KR259574 |
| UM 0015 | KR259578 |
| UM 0016 | KR259580 |
| UM 0018 | KR259583 |
| UM 0020 | KR259584 |
| UM 0021 | KR259587 |
| UM 0029 | KR259589 |
| UM 0032 | KR259592 |
| UM 0034 | KR259594 |
| UM 0047 | KR259597 |
| UM 0050 | KR259598 |
| UM 0058 | KR259601 |
| UM 0060 | KR259604 |
| UM 0063 | KR259608 |
| UM 0070 | KR259614 |
| UM 0088 | KR259615 |
| UM 0090 | KR259617 |
| UM 0092 | KR259620 |
| UM 0105 | KR259623 |
| UM 0115 | KR259628 |
| UM 0118 | KR259632 |
